# Retraction: No Mow May lawns have higher pollinator richness and abundances: An engaged community provides floral resources for pollinators

**DOI:** 10.7717/peerj.10021/retraction

**Published:** 2022-11-18

**Authors:** PeerJ Editorial Office

Retraction: Del Toro I, Ribbons RR. 2020. No Mow May lawns have higher pollinator richness and abundances: An engaged community provides floral resources for pollinators. PeerJ 8:e10021 https://doi.org/10.7717/peerj.10021

After finding several potential inconsistencies in data handling and reporting, the authors and editorial team have agreed to retract this article with the opportunity for re-evaluation should the authors choose to submit a new version.

PeerJ Editorial Office. 2022. Retraction: No Mow May lawns have higher pollinator richness and abundances: An engaged community provides floral resources for pollinators. PeerJ 8:e10021/retraction https://doi.org/10.7717/peerj-e10021/retraction.

